# The Brain Metabolome of Male Rats across the Lifespan

**DOI:** 10.1038/srep24125

**Published:** 2016-04-11

**Authors:** Xiaojiao Zheng, Tianlu Chen, Aihua Zhao, Xiaoyan Wang, Guoxiang Xie, Fengjie Huang, Jiajian Liu, Qing Zhao, Shouli Wang, Chongchong Wang, Mingmei Zhou, Jun Panee, Zhigang He, Wei Jia

**Affiliations:** 1 Shanghai Key Laboratory of Diabetes Mellitus and Center for Translational Medicine, Shanghai Jiao Tong University Affiliated Sixth People’s Hospital, Shanghai 200233, China; 2 Ministry of Education Key Laboratory of Systems Biomedicine, Shanghai Center for Systems Biomedicine, Shanghai Jiao Tong University, Shanghai 200240, China; 3 University of Hawaii Cancer Center, Honolulu 96813, USA; 4 Center for Chinese Medical Therapy and Systems Biology, E-Institute, Shanghai University of Traditional Chinese Medicine, Shanghai 201203, China; 5 F. M. Kirby Neurobiology Center, Children’s Hospital, and Department of Neurology, Harvard Medical School, Boston, MA 02115, USA

## Abstract

Comprehensive and accurate characterization of brain metabolome is fundamental to brain science, but has been hindered by technical limitations. We profiled the brain metabolome in male Wistar rats at different ages (day 1 to week 111) using high-sensitivity and high-resolution mass spectrometry. Totally 380 metabolites were identified and 232 of them were quantitated. Compared with anatomical regions, age had a greater effect on variations in the brain metabolome. Lipids, fatty acids and amino acids accounted for the largest proportions of the brain metabolome, and their concentrations varied across the lifespan. The levels of polyunsaturated fatty acids were higher in infancy (week 1 to week 3) compared with later ages, and the ratio of omega-6 to omega-3 fatty acids increased in the aged brain (week 56 to week 111). Importantly, a panel of 20 bile acids were quantitatively measured, most of which have not previously been documented in the brain metabolome. This study extends the breadth of the mammalian brain metabolome as well as our knowledge of functional brain development, both of which are critically important to move the brain science forward.

The brain is the center of the nervous system, communicating with the other organs in the body, and controlling motor, sensory, and cognitive functions. Proper function of the brain relies on strictly regulated metabolism within this complex organ, as disturbances in brain metabolism are associated with several neurological diseases, such as Alzheimer’s disease^1^,^2^, Parkinson’s disease^3^, and schizophrenia^4^. Brain metabolism is also tightly regulated during brain development, maturation and aging.

Metabolomics is a powerful platform for systematic metabolite profiling in a biological system^5^, such as in the brain. The brain metabolome represents all compounds that are involved in brain development and function, including membrane lipids, building blocks of proteins and polysaccharides, neurotransmitters, and other biologically active compounds^6^,^7^. Previous brain metabolomic studies have been performed on tissues collected from humans, chimpanzees, rhesus macaques^8^,^9^, rats^10^,^11^, and mice^9^,^12^. These studies have identified energy and neurotransmission metabolites and changes associated with neurological diseases and mammalian evolution^8^,^10^,^13^. However, the number of metabolites identified to date has been relatively small, and the majority of the detected metabolites were not quantitated, due to technical limitations^8^,^9^. More comprehensive and accurate analytical methods are needed to improve our knowledge of the brain metabolome.

In the present study, chromatography coupled to mass spectrometry was applied to profile the brain metabolome in male Wistar rats to determine age and region related metabolome variations. We detected most metabolites that have been previously reported, such as lipids, free fatty acids and amino acids. More importantly, using a protocol optimized for bile acid detection, we were able to quantitate 20 bile acids in the rat brain, most of which have not previously been documented in the brain metabolome.

## Results

### Overview of the rat brain metabolome

Wild type male Wistar rats were sacrificed on postnatal days 1 (D1), 7 (week 1, W1), 21 (week 3, W3), 49 (week 7, W7), 63 (week 9, W9), 84 (week 12, W12), 168 (week 24, W24), 392 (week 56, W56), and 777 (week 111, W111). Whole brains were collected at all 9 time points. In addition, three anatomical regions, the cerebral cortex, hippocampus and thalamus, were also collected from W7 to W111. Six (6) rats were used to collect whole brain samples and 6 rats were used for regional samples at each time point. The body and brain weights at the time of sacrifice are shown in Supplementary Fig. S1.

**Figure 1:**
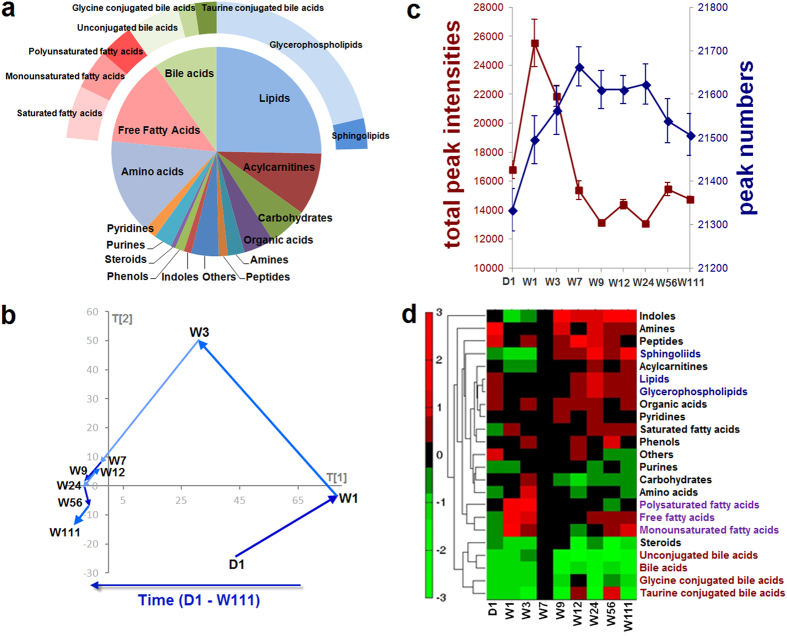
Brain metabolome overview and its age related variation. (**a**) Pie chart shows 15 metabolite types and subtypes in the brain metabolome. Free fatty acids (green) include saturated fatty acids, monounsaturated fatty acids, and polysaturated fatty acids; lipids (blue) include glycerophospholipids and sphingolipids; bile acids (red) include unconjugated bile acids, glycine conjugated bile acids and taurine conjugated bile acids. (**b**) The metabolome trajectory shows the overall fluctuation with age based on multivariate statistics principal component analysis (PCA) modeling. The value of each point in the x-axis and y-axis indicates the mean value of principal component 1 (PC 1) and principal component 2 (PC 2), respectively, at each time point. (**c**) The line chart shows the mean values of total metabolite intensities (red) and detected peak numbers (blue) at different time points. The error bars indicate standard error. (**d**) Hierarchical cluster analysis (HCA) with Euclidean distance was generated based on fold change values of mean concentrations at each time point compared with W7. In the dendrogram, lipids and alcycarnitines are clustered in the blue node, free fatty acids are clustered in the purple node, and bile acids are clustered in the red node.

Three metabolomic platforms were used for metabolite identification and quantitation: ultra-performance liquid chromatography coupled to triple quadrupole mass spectrometry (UPLC-TQMS), ultra-performance liquid chromatography coupled to quadrupole time-of-flight mass spectrometry (UPLC-QTOFMS), and gas chromatography coupled to time-of-flight mass spectrometry (GC-TOFMS). A total of 22,788 peaks were detected, some of which presented in a single individual sample. We identified 380 metabolites based on our in-house standard library and online available libraries, and 232 were quantitated (Supplementary Dataset). The brain metabolome consisted of a wide variety of metabolites, including lipids, free fatty acids, amino acids, and bile acids. We further separated some metabolite types into subtypes, for instance, (1) total bile acids were separated into unconjugated bile acids, glycine conjugated bile acids and taurine bile acids, (2) total free fatty acids were separated into saturated fatty acids, monounsaturated fatty acids, and polyunsaturated fatty acids, and (3) total lipids were separated into glycerophospholipids and sphingolipids. The proportions of the metabolite types and subtypes in the metabolome are shown in Fig. 1a, and the total numbers of metabolites identified and quantitated under each type and subtype are listed in Supplementary Table S1.

### Age-related changes in the brain metabolome

Age-related brain metabolome alterations were evaluated based on the score values of multivariate statistics principal component analysis (PCA) models. Figure 1b shows the age-associated metabolome trajectory. The brain metabolome shifted significantly during the infancy (D1 to W3) and adolescence (W3 to W7). During adulthood (W7 to W56) the metabolome trajectory was relatively steady, with only slight variations. The trajectory of old age (W56 to W111) shifted to a small extent.

These age-related brain metabolome alterations were further evaluated using peak numbers and total peak intensity of the metabolites. As shown in Fig. 1c, metabolite peak numbers gradually increased over the first seven weeks, remained stable from W7 to W24, and decreased from W24 to W111. The total metabolite intensity reached a peak value at W1, decreased gradually from W1 to W7, and remained constant from W7 to W111. These results highlight the age-related metabolic features of the brain: (1) At W1, while the peak number was still increasing, the total peak intensity reached the highest value, indicating that large quantities of certain metabolites might be required for early brain development. (2) At the end of adolescence (W7), the number of metabolites reached a plateau, but the peak intensity reached a nadir, indicating that although the maximum number of metabolites was detected, only a few of these compounds were required in large amount at this stage of brain maturation. (3) The pattern observed at W7 remained unchanged during young adulthood (W7–W24). (4) During aging (W24–W111), the number of detectable metabolites started to decrease. Both Fig. 1b,c illustrate the significant brain metabolome alterations that occur during infancy, adolescence, and aging, with relatively few changes during adulthood.

Changes in the concentration changes of each type and subtype of metabolites were assessed using hierarchical cluster analysis (HCA) with Euclidean distance generated based on the fold change values (Fig. 1d). Table 1 shows these concentration changes relative to age. W7 was used as a reference point because the overall metabolite concentration seemed to have reached a steady state at this age. Some types of metabolites had similar trends of variation across different ages, and therefore could be clustered to one node. These metabolites included bile acids and associated subtypes, fatty acids and two subtypes of unsaturated fatty acids as well as lipids and acylcarnitines.

### Region-related changes in the brain metabolome

**Figure 2:**
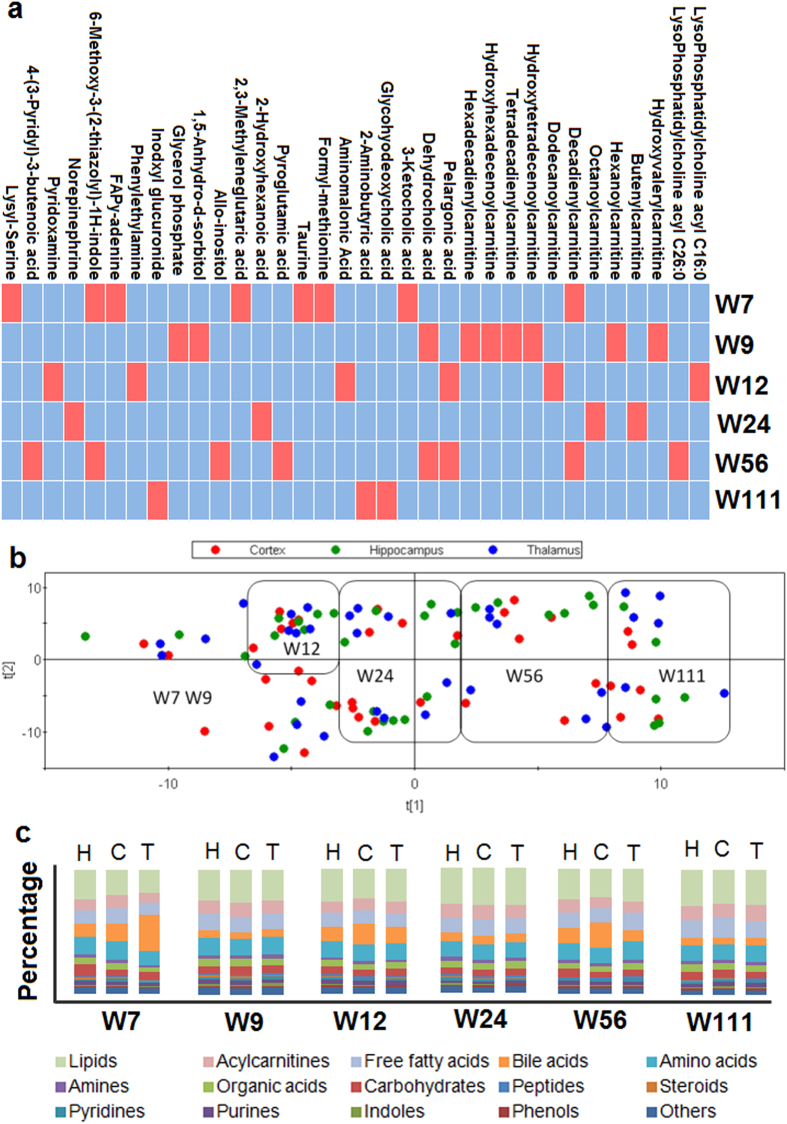
Regional variation of brain metabolome and comparison between age- and region-related variations. (**a**) The grid diagram shows the metabolites with significant differences among the three brain regions at any time point. The red blocks indicate statistically significant differences (Kruskal-Wallis test, *P* < 0.05), and the blue blocks indicate no significant difference (Kruskal-Wallis test, *P* > 0.05). (**b**) Partial Least Squares Projection to Latent Structures (PLS) scores plot was generated based on the data from the three regions at different time points. This analysis shows clear discrimination by age, with little obvious regional discrimination. (**c**) The bar plots show the metabolite types of the three brain regions at different time points. H, hippocampus; C, cortex; T, thamalus.

Multivariate statistics partial least squares-discriminate analysis (PLS-DA) was used to assess regional metabolomic changes between the cerebral cortex, thalamus, and hippocampus at each time point, but no model could be constructed. The univariate statistics Kruskal-Wallis test was then carried out to assess differences in individual metabolite concentrations among the three regions at any time point. Significant differences were found for 34 metabolites (*P* < 0.05) (Fig. 2a), most of which were acylcarnitines. We further compared differences in metabolite type and subtype concentrations between any two of the three regions at each time point using the Mann Whitney test (Table 2); significant differences were found in aclycarnitines, amines and peptides. As shown in Table 2, significant differences were observed only when the cortex was compared with the hippocampus or thalamus, not when the hippocampus and thalamus were compared with each other.

### Comparison of age- and region- related changes in the brain metabolome

Principle variance component analysis (PVCA)^8^ was used to evaluate the weight of different contributing factors to the changes in the brain metabolome. As shown in Supplementary Fig. S2, 43.58% of the total metabolic variation was explained by age, 16.12% by body weight, 1.81% by brain region, 0.06% by brain weight, and 38.43% by other residual factors. Metabolomic differences across age and brain regions were further visualized using Partial Least Squares Projection to Latent Structures (PLS) models (Fig. 2b). Consistent with the PVCA results, distinct metabolome patterns were observed in the time series, but not among the three brain regions at a single time point. The percentages of metabolite types in each region at different time points are also shown in Fig. 2c, although regional variations can be seen (such as those for bile acids at W7, W12 and W56), most of the variations were not statistically significant (Table 2). Therefore, compared with regional differences, age contributed more substantially to the changes in the brain metabolome.

### Age-related changes in major metabolites in the brain metabolome

**Figure 3:**
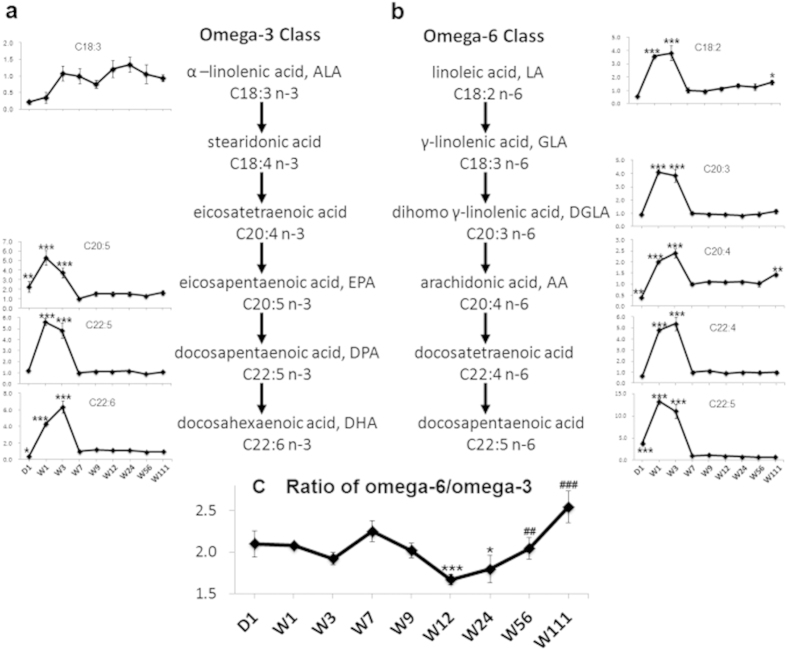
Dynamic alterations in omega-3 and omega-6 polyunsaturated fatty acids across the lifespan. (**a**) The line charts show the fold changes (y axis) of each omega-3 fatty acid along the metabolic pathway as the ratio of the concentration of each time point to that of W7 (x axis). (**b**) The line charts show the fold changes (y axis) of each omega-6 fatty acid along the metabolic pathway as the ratio of the concentration of each time point to that of W7 (x axis). (**c**) The line chart shows the changes in the total omega-6 to omega 3 concentration ratio (y axis) across different time points (x axis). The error bars indicate standard errors. N = 6 for each group. *indicates *P* < 0.05, **indicates *P* < 0.01, and ***indicates *P* < 0.001 compared with W7 in Mann Whitney tests. ^##^indicates *P* < 0.01, and ^###^indicates *P* < 0.001 compared with W12 in Mann Whitney tests.

As shown in Table 1, the concentrations of total free fatty acids and associated subtypes were significantly higher during infancy (W1 and W3) and senescence (W111) compared with W7, while the concentrations at other time points were similar to W7. Polyunsaturated fatty acids were further categorized into omega-3 and omega-6 fatty acids based on their chemical structures. Figure 3a,b show that both omega-3 and omega-6 fatty acids, except for alpha-linolenic acid (ALA), were significantly higher during infancy (W1 and W3) than at W7. Two omega-6 fatty acids, linoleic acid (LA) and arachidonic acid (AA), were also increased at W111 compared with W7. No such increase was observed in the omega-3 class. Figure 3c shows that the concentration ratios between the omega-3 and omega 6 classes remained constant from D1 to W9, were the lowest at W12 and W24, and then gradually increased to reach the highest level at W111.

Table 1 and Supplementary Fig. S3 also show that the concentrations of lipids were significantly higher at all other time points (except for W9) compared with W7. Lipids were further categorized into glycerophospholipids and sphingolipids. As glycerophospholipids made up the larger portion (86.14%) of lipids, their age-dependent variation was in line with that of the lipids. In contrast, sphingolipids, which comprised the smaller portion of the lipids, were present at lower levels during the first three weeks and at higher levels from W9 to W111 compared with W7.

**Figure 4:**
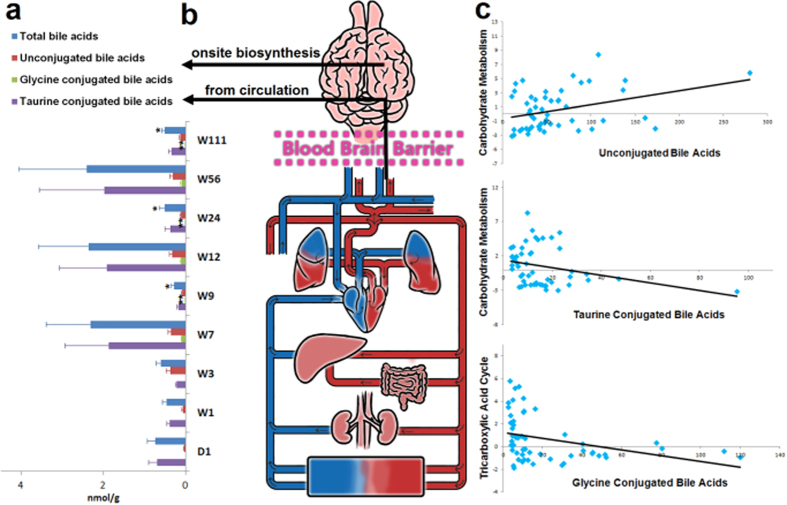
Bile acids in the brain. (**a**) The bar chart shows the concentrations of total bile acids and three bile acid subtypes at different time points. The error bars indicate standard error. N = 6 for each group. *indicates *P* < 0.05, and **indicates *P* < 0.01 compared with W7 in Mann Whitney test. (**b**) The figure shows two possible sources of brain bile acids, i.e., blood circulation and onsite biosynthesis in the brain. (**c**) The scatter plots generated by Spearman correlation coefficient analysis illustrate that unconjugated bile acids were positively correlated with carbohydrate metabolism, and taurine conjugated bile acids and glycine conjugated bile acids were inversely correlated with carbohydrate metabolism and the tricarboxylic acid cycle, respectively.

Importantly, 20 bile acids were also detected and quantitated in the rat brain, including 9 unconjugated bile acids, 4 glycine conjugated bile acids and 7 taurine conjugated bile acids. The age-related changes in total bile acids and associated subtypes are shown in Fig. 4a. The two possible sources of bile acids in the brain are shown in Fig. 4b. Furthermore, the total concentrations of bile acids and their subtypes were correlated with selected metabolites in carbohydrate metabolism and the tricarboxylic acid cycle (Supplementary Table S2). Figure 4c shows that unconjugated bile acids positively correlated with carbohydrate metabolism and that taurine- and glycine-conjugated bile acids negatively correlated with carbohydrate metabolism and the tricarboxylic acid cycle, respectively.

## Discussion

Comprehensive and accurate characterization of the brain metabolome is fundamental to brain science. Taking full advantage of the available high-resolution and high-sensitivity mass spectrometry (MS) instruments, we combined several MS platforms to maximize the size of the metabolome detected. Additionally, we used our in-house library, which contains more than 1,000 metabolite standards, to annotate the detected metabolites, resulting in a panel of 380 well-annotated brain metabolites, with 232 accurately quantitated. More importantly, using an optimized protocol for bile acids, we were able to identify and quantitate bile acids that have not previously been documented in the brain metabolome.

Dynamic neurodevelopment and substantial biochemical changes have been observed in the brain during infancy and adolescence^14^. Accordingly, our study showed that most of the changes in the brain metabolome occurred during this age range. For example, the total peak intensity was the highest at W1, which coincided with the peak concentrations of glycerophospholipids, free fatty acids (especially unsaturated free fatty acids), amino acids, organic acids, carbohydrates, phenols, and pyridines occurring at this time point (Table 1). These metabolites are required by energy metabolism and also function as structural building blocks to support brain development. A previous study reported that during early postnatal brain development, neurons increase in size, elaborate their terminal dendritic and axonal arborizations, and form connections^15^; we also observed during infancy higher levels of certain amino acid neurotransmitters (Supplementary Fig. S4), such as 4-aminobutyric acid, glycine, aspartic acid and glutamic acid, which may biochemically contribute to synapse formation and neurotransmission at this age. On the other hand, the aged brain also undergoes significant biochemical, cellular and structural changes; these changes are frequently associated with declines in brain functions, such as impaired learning and memory^16^. The change in the trajectory from W56 to W111 demonstrates an altered developmental pattern compared with that of early ages (W3–W7). The concentrations of some neurologically active amino acids, such as 4-aminobutyric acid, glutamic acid, and taurine decreased (Supplementary Fig. S4), while other neurologically harmful compounds, such as amines and indoles increased (Table 1) in the aging or aged brain.

The cerebral cortex, hippocampus and thalamus have reciprocal interconnections and serve well recognized roles in memory processing^17^,^18^,^19^. Several age-related changes in these brain regions were previously reported, including decreases in regional volume^20^, loss of synapse, reduction of dendritic spine density^21^, and alteration of gene expression^22^,^23^. Table 2 shows that the cortex exhibited metabolic differences relative to the hippocampus and thalamus, especially during old age (W56 and W111), while the hippocampus and thalamus remained metabolically similar across all time points. Acylcarnitines and amines both contributed to the regional differences. Acylcarnitines are involved in the transportation of long-chain acyl groups from fatty acids into the mitochondrial matrix, and play an important role in energy metabolism^24^. In the aged brain (W56 and W111), the concentrations of acylcarnitines were significantly lower in the cortex compared with the hippocampus and thalamus. Our study also revealed that brain region-related variation was much smaller than age-related variation in the brain metabolome at the time points examined, suggesting that the age-related changes might occur in the three regions simultaneously (Supplementary Fig. S5). A limitation of the study is that the separated brain regions were not collected from rats younger than 7 weeks, and the above conclusion therefore cannot be extrapolated to infancy and adolescence, when the brain development is the most active. Future studies are needed to delineate regional variations in the brain metabolome during the early periods of brain development.

Free fatty acids are major structural components in the brain^25^. Both omega-3 and omega-6 polyunsaturated fatty acids have important roles in neural development and aging^26^. The brain starts accumulating polyunsaturated fatty acids in utero, continuing during the first few weeks of neonatal brain growth^27^, when increases in glial cells, outgrowth of axons and dendrites, and myelination of nerve fibers occur^28^,^29^. Our results (Fig. 3) showed high concentrations of omega-3 and omega-6 polyunsaturated fatty acids in the brain during infancy (W1–W3), indicating a high demand for these “building materials” by rapid neural development. In contrast to the other polyunsaturated fatty acids, the concentration of alpha-linolenic acid was the lowest at D1 and W1; this might be due to the high conversion rate of this compound to downstream metabolites during the three prenatal weeks. Omega-3 and omega-6 fatty acids can also be converted to eicosanoids, which are signaling molecules that regulate inflammation^30^. Typically omega-6 fatty acids have proinflammatory properties, whereas omega-3 fatty acids exert anti-inflammatory effects^31^,^32^. Our results showed a gradual increase in the omega-6 to omega-3 ratio from W24 to W111, which may reflect the onset of neuroinflammation during brain aging. Our data also revealed some omega-6 fatty acids, such as docosapentaenoic acid and cis-10,13-nonadecadienoic acid, showed downward trends in the older rats (Supplementary Fig. S6). We will investigate the different patterns of age-related variation among omega-6 fatty acids in further studies.

The nervous system is highly enriched in lipids^33^. We measured two types of lipids in this study, namely glycerophospholipids (mainly phosphatidylcholine (PC) and lysophosphatidylcholine (lysoPC)) and sphingolipids (mainly sphingomyelins) (Supplementary Fig. S3). Glycerophospholipids form the essential lipid bilayer of biological membranes and are intimately involved in signal transduction, regulation of membrane trafficking and many other membrane-related phenomena^34^,^35^,^36^. LysoPC is derived from PC via phospholipase A2-mediated partial hydrolysis^37^. Alteration of membrane glycerophospholipid composition in neurons has been associated with cognitive disorders^6^, and the plasma PC to lysoPC ratio can also differentiate healthy controls from patients with Alzheimer’s disease or mild cognitive impairments^38^. Our study (Supplementary Fig. S3) detected different patterns of PC and lysoPC alterations in the whole brain at different time points, which might reflect differences in phospholipase A2 activity across the lifespan. Sphingolipids also mediate signal transduction in cells^33^, regulate neuronal differentiation and survival, and synchronize the development of the nervous system^39^. These metabolites showed differences across ages, most with low levels during infancy. Furthermore, the catabolism of sphingomyelins (a subtype of sphingolipids) generates multiple bioactive lipids, such as sphingosine, sphinganine and phytosphingosine^39^. Increased sphingomyelins content has been reported in the aged brain^40^, and studies on human and animal tissues and cultured cells have supported the involvement of perturbed sphingomyelin metabolism in several neurodegenerative disorders^39^. The low levels of sphingomyelins present at infancy, as shown in our study, might imply the upregulation of sphingomyelin catabolism during this period of brain development.

Importantly, using our optimized bile acid quantitative method, we were able to quantitate 20 bile acids in the rat brain. Except for cholic acid, chenodeoxycholic acid, deoxycholic acid, and ursodeoxycholic acid and their conjugated types, which have been reported before^41^, most of these bile acids have not previously been detected in the brain. The possible sources of bile acids in the brain are not yet clear. As we previously detected most of these 20 bile acids in rat blood^42^, it is possible that the bile acids in the brain are transported across the blood–brain-barrier from the circulation^43^,^44^. This notion is further supported by the fact that Ostα-Ostβ, the bile acid transporters that are critical for the intestinal absorption and the enterohepatic circulation of bile acids^45^, are also expressed in the brain^46^. On the other hand, the enzymes (CYP8B1, CYP27A1)^47^,^48^ and the precursor compound (24S-hydroxycholesterol)^49^ required for bile acid synthesis have also been found in the brain, suggesting that some proportion of these bile acids might be synthesized in the brain (Fig. 4b). Bile acids are signaling molecules with broad paracrine and endocrine functions^50^. The bile acid receptors FXR and TGR5, which are associated with glucose and energy homeostasis^46^,^51^, have been found in the central nervous system^52^. In line with these findings, we show that both unconjugated bile acids and taurine conjugated bile acids correlated with carbohydrate metabolism, though with inverse correlation coefficients, and that glycine conjugate bile acids positively correlated with energy metabolism (tricarboxylic acid cycle) in the brain (Fig. 4c). More importantly, bile acids act as neuroactive steroids in the brain^53^. Different classes of bile acids can either inhibit or potentiate GABA_A_ or NMDA receptors^53^. Decreased levels of bile acids or their intermediates have been associated with neurodegenerative disorders, such as Alzheimer’s and Parkinson’s disease^54^,^55^. Ursodeoxycholic acid and its taurine-conjugate, tauroursodeoxycholic acid, have been reported to exert neuroprotective effects, acting as potent inhibitors of apoptosis in neurons, and hence have therapeutic potential in treating neurological disorders^56^,^57^. Our study demonstrated that bile acids are significant components of the brain, and further studies are warranted to understand the function of these compounds in the brain under both physiological and pathogenic conditions.

In summary, this comprehensive metabolomic study identified nearly 400 metabolites in the brain of male Wistar rats, and more than half were accurately quantitated. Age explain more of the variations in the brain metabolome than specific anatomical regions. In addition to known brain metabolites such as lipids, free fatty acids and amino acids, we also detected and quantitated 20 bile acids in the rat brain, the majority of which have not been documented previously.

## Methods

### Animal experiments

Wistar rats (4 weeks old) purchased from Shanghai Laboratory Animal Co Ltd. (SLAC, Shanghai, China) were housed in a specific-pathogen-free (SPF) environment under a controlled 12 h light/12 h dark cycle at 20–22 °C and 45 ± 5% humidity, with free access to sterilized chow and water. Male and female rats were raised separately. At 8 weeks of age, female and male rats were paired. From 20 days after pairing, all pairs were monitored daily for signs of birth. The day of birth was defined as day 0 for each litter. The gender of mice was determined through observation, as well as gender specific *Sry* gene^58^. For the rats of one week old or older than one week, male and female were differentiated by observing the distance from the anus and the genital papilla (the anogenital distance), which is greater in males. The example of the distances in the rats of 1-week and 3-week old were shown in Supplementary Fig. S7. For 1-day old rats, it was really hard to determine through observation. We applied a fast sex identification approach using PCR amplification of male-specific *Sry* gene. We amplified fragments of duplex PCR reaction using HMG-SRY-primers (5′–3′) GTC AAG CGC CCC ATG AAT GCA T and (3′–5′) AGT TTG GGT ATT TCT CTC TGT G. The results could be visualized by electrophoresis in 1.5% ethidiumbromid-stained agarose gel. Amplification of the 202-bp *Sry* fragment was successful only in males (Supplementary Fig. S8).

The body weights of the rats were measured before sacrifice at postnatal D1, W1, W3, W7, W9, W12, W24, W56 and W111. The whole brain (D1 to W111) as well as three anatomical regions, cerebral cortex, thalamus, and hippocampus (W7 to W111), were collected and weighed. To reduce the influences of the postmortem delay on the brain metabolites, all samples were collected and frozen at −80 °C within 10 min after death. At each time point, 6 rats were sacrificed to collect whole brain samples and 6 rats were sacrificed to collect samples from the specific brain regions.

All animal handling and experiments were performed strictly in accordance with the recommendations of the Guide for the Care and Use of Laboratory Animals of the National Institutes of Health. The experimental protocol was approved by the Center for Laboratory Animals, Shanghai Jiao Tong University, Shanghai, China.

### Sample treatment

Approximately 50 mg of each tissue sample was weighed and extracted in a two-step extraction. First, 75 μL of a precooled solvent mixture of methanol and water (1:1) was added, and the sample was homogenized for 3 min followed by centrifugation at 12,000 *g* for 15 min. The supernatant was transferred into a new tube. The residue was extracted with 200 μL of a precooled methanol and chloroform mixture (3:1) using a homogenizer followed by centrifugation. The supernatant was combined with the previous extraction for subsequent metabolite (except for free fatty acids) extraction. Pooled quality control (QC) samples were prepared by mixing 20 μL of each sample extraction.

Metabolite profiling was performed using two instrumental platforms, UPLC-QTOFMS and GC-TOFMS. For UPLC-QTOFMS analysis, the internal standard (20 μL of p-chlorophenylalanine in water, 30 μg/mL) was added to 50 μL of sample extraction, vortexed, and then vacuum dried. The residue was reconstituted with 80 μL of a solvent mixture of acetonitrile and water (9:1) for analysis. For GC-TOFMS analysis, the internal standards (10 μL of p-chlorophenylalanine in water, 0.1 mg/mL; 10 μL of heptadecanoic acid in methanol, 1 mg/mL) were added to 50 μL of sample extraction, vortexed, and vacuum dried for TMS derivatization prior to analysis, following the procedure developed by our lab.

The AbsoluteIDQ^TM^ p180 Kit (BIOCRATES Life Sciences AG, Innsbruck, Austria) was applied for quantitation of lipids, acylcarnitines, amino acids, biogenic amines and sugars. Sample extraction was perfomed according to the user manual. Briefly, 10 μL of sample extraction and 10 μL of internal isotope-labeled standards were added to the 96-well plate and dried for 30 min under nitrogen flow. Phenylisothiocyanate (5%, v/v) was then added for derivatization with 20-min incubation and 45-min drying under nitrogen flow. A 300-μL aliquot of extraction solvent (5 mM ammonium acetate in methanol) was added, and shaken for 30 min at 450 *g*. The samples were filtered by centrifugation for 2 min at 50 *g*, and separated into two parts. A 150-μL aliquot of the filtrate was transferred to a new 96-well plate and diluted with 150 μL water for subsequent UPLC/TQMS analysis; 100 μL of the remainder was diluted with 500 μL MS running buffer for flow injection (FIA) analysis.

For quantitation of bile acids, 100 μL of sample extraction was mixed with 10 μL of internal standard (containing 1 μM of cholic acid-D4, ursodeoxycholic acid-D4, lithocholic acid, glycocholic acid-D4 and glycodeoxycholic acid-D4) and vacuum-dried. The residue was redissolved with an equal amount of acetonitrile and methanol (19:1) with 0.1% formic acid and water with 0.1% formic acid to a final volume of 50 μL. After centrifugation, the supernatant was used for analysis.

For quantitation of fatty acids, approximately 50 mg of each tissue samples was weighed and extracted with 500 μL of isopropanol and hexane (4:1) with 2% phosphate (2 M), and then homogenized for 3 min followed by centrifugation at 12,000 *g* for 10 min. The supernatant (450 μL) supernatant was transferred into a new tube. The internal standard (10 μL of nonadecylic acid-D37, 60 μM), 800 μL of hexane and 300 μL of water were then added, and the mixture was vortexed for 2 min and centrifuged for 10 min at 12,000 *g*. The supernatant (800 μL) was transferred to a new tube and dried under vacuum. The residue was reconstituted with 1,400 μL of methanol and subjected to analysis.

### Metabolite measurement

Metabolite measurement was performed using established instrumental methods modified for application to rat brain. All samples were injected in randomized order to minimize systematic analytical deviations.

Metabolite profiling was performed by two instrumental platforms, UPLC/QTOFMS (Xevo G2, Waters Corp., Milford, MA) and GC/TOFMS (Pegasus HT, Leco Corp., St. Joseph, MI), using our previous instrumental parameters with minor optimization. A pooled extraction QC sample was injected every ten sample injections. Spectral data analysis was performed by Markerlynx and ChromaTOF software. Compound identification was performed using our in-house library containing over 1,000 mammalian metabolite standards as well as available online libraries, such as the Human Metabolome Database (HMDB) and the National Institute of Standards and Technology (NIST).

The AbosoluteIDQ p180 Kit assay was performed by a combined FIA and UPLC/TQMS (Xevo G2, Waters Corp., Milford, MA) with an electrospray ionization source. The assay workflow was performed following the Biocrates software, from sample registration to data processing. Calibration curves were generated using 40 different chemical standards at 7 different concentration levels. Because not all of the lipid standards were commercially available, the semi-quantitation was applied in the lipid quantitation based on isotopic lipid internal standards. One series of standard calibration curve was analyzed every 89 sample injections.

Bile acid analysis was performed by UPLC/TQMS. The elution solvents were water+0.01% formic acid (A) and acetonitrile+0.01% formic acid (B). The elution gradient over 20 min at a flow rate of 450 μL/min was as follows: 0–2 min (20% B), 2–3 min (20–25% B), 3–6 min (25% B), 6–8 min (25–35% B), 8–11.5 min (35% B), 11.5–18 min (35–99% B), 18–19 min (99% B), and 19–20 min (99–20% B). The MS was operated at a negative electrospray ionization mode. The cone and collision energy for each bile acid used the optimized settings from the QuanOptimize application manager (Waters Corp., Milford, MA). One standard calibration solution at 10 different concentration levels contains 45 standards and was tested every 40 samples. Peak annotation and quantitation were performed by TargetLynx application manager (Waters Corp., Milford, MA).

Free fatty acids were analyzed by UPLC/QTOFMS. The elution solvents were water (A) and acetonitrile/isopropanol (v/v = 80/20, B) with a flow rate of 400 μL/min. The initial gradient was 70% B and maintained for 2 min, increased to 75% B over 3 min, increased to 80% over 5 min, increased to 90% over 3 min, increased to 99% over 3 min and kept at 99% for 5 min before switching back to the initial condition. The MS was operated at a positive electrospray ionization mode. One standard calibration solution with 65 free fatty acid standards at 10 different concentration levels was analyzed every 40 sample injections. Peak annotation and quantitation were performed by TargetLynx application manager (Waters Corp., Milford, MA).

### Statistical analysis

The metabolite profiling data were adjusted by pooled QC using the Metabolic Data Calibration Tool to eliminate systematic deviations. Missing values (less than 1%) were replaced by mean values of corresponding groups. The fold changes were the concentration or intensity mean value ratios of two groups. HCA using Euclidean distance was generated based on the fold change values of mean concentration values at each time point compared with W7. Univariate statistical analysis, non-parametric Wilcoxon Mann Whitney test and Kruskal-Wallis test were performed in SPSS (IBM, USA, V22), with *P* < 0.05 set as the level of statistical significance. Multivariate statistical analysis, PCA and PLS-DA were carried out in Simca-P+ (Umetrix, Sweden, V12.0). The metabolome trajectory was constructed based on the mean values of the first 2 principal components scores at each time point from the PCA models. The total concentrations of bile acids and three subtypes were correlated with the first principal component scores of the PCA derived from the metabolites of carbohydrate metabolism or the tricarboxylic acid cycle by calculating their Spearman’s correlation coefficients in SPSS. PVCA was applied in R to calculate the variance explained by age, body weight, and brain region (detailed method in Supplementary Methods) (Supplementary Fig. S9).

## Additional Information

**How to cite this article**: Zheng, X. *et al*. The Brain Metabolome of Male Rats across the Lifespan. *Sci. Rep.* **6**, 24125; doi: 10.1038/srep24125 (2016).

## Supplementary Material

Supplementary Information

Supplementary Dataset

## Figures and Tables

**Table 1:** The concentration variations of metabolite types and subtypes along age (N = 6 each group).

**Metabolite Types**	**Fold Change**								**P Value (Mann Whitney test)**							
	**D1/W7**	**W1/W7**	**W3/W7**	**W9/W7**	**W12/W7**	**W24/W7**	**W56/W7**	**W111/W7**	**D1 vs W7**	**W1 vs W7**	**W3 vs W7**	**W9 vs W7**	**W12 vs W7**	**W24 vs W7**	**W56 vs W7**	**W111 vs W7**
**Lipids**	1.57	1.71	1.19	1.03	1.10	1.20	1.15	1.29	3.30E-05	3.05E-07	1.97E-02	5.47E-01	3.54E-02	8.07E-04	8.63E-03	3.90E-05
Glycerophospholipids	1.75	1.90	1.28	1.01	1.11	1.18	1.13	1.20	6.35E-06	4.57E-08	3.55E-03	8.13E-01	4.71E-02	4.73E-03	3.67E-02	2.91E-03
Sphingolipids	0.56	0.39	0.49	1.19	1.03	1.37	1.26	1.84	7.36E-07	2.20E-09	3.12E-08	1.15E-02	6.69E-01	9.24E-06	1.95E-05	1.06E-10
**Acylcarnitine**	1.15	0.98	0.95	1.03	0.95	1.06	1.07	1.33	4.79E-02	8.36E-01	4.89E-01	6.03E-01	3.13E-01	3.17E-01	2.22E-01	4.00E-04
**Free Fatty Acids**	0.85	4.26	2.18	1.01	0.93	0.99	1.17	1.33	4.51E-01	5.00E-03	1.82E-05	9.42E-01	5.56E-01	9.47E-01	3.70E-01	3.95E-02
Saturated fatty acids	0.72	2.92	1.45	1.01	1.01	1.04	1.26	1.33	7.08E-02	4.36E-02	7.36E-03	9.59E-01	9.41E-01	6.76E-01	9.10E-02	1.08E-02
Monounsaturated fatty acids	0.82	6.01	1.70	1.06	0.80	0.95	1.22	1.60	4.26E-01	3.27E-02	9.78E-03	6.97E-01	1.54E-01	7.45E-01	3.55E-01	1.07E-02
Polyunsaturated fatty acids	1.10	4.51	3.82	0.96	0.93	0.96	0.97	1.05	7.00E-01	2.69E-12	1.32E-08	8.35E-01	6.76E-01	7.90E-01	9.00E-01	7.44E-01
**Bile acids**	0.27	0.30	0.56	0.27	0.69	0.32	0.75	0.30	1.87E-01	2.06E-01	4.18E-01	2.25E-02	3.43E-01	1.33E-02	5.21E-01	1.10E-02
Unconjugated bile acids	0.25	0.34	0.73	0.28	0.50	0.31	0.50	0.30	3.25E-01	3.86E-01	7.21E-01	9.86E-02	1.89E-01	6.91E-02	2.49E-01	6.23E-02
Glycine conjugated bile acids	0.36	0.37	0.34	0.41	1.01	0.45	0.95	0.44	7.05E-02	7.48E-02	6.08E-02	5.98E-03	9.80E-01	2.57E-03	8.66E-01	1.74E-03
Taurine conjugated bile acids	0.27	0.14	0.19	0.14	1.04	0.23	1.34	0.20	4.30E-01	3.50E-01	3.79E-01	1.03E-01	9.61E-01	9.15E-02	7.60E-01	8.05E-02
**Amino acids**	0.81	2.09	2.36	0.87	0.91	0.81	1.01	0.89	1.85E-01	6.35E-07	5.83E-08	1.19E-01	2.27E-01	1.24E-02	9.50E-01	2.39E-01
**Organic acids**	1.50	1.85	1.75	1.15	1.02	1.03	1.00	1.24	8.22E-04	1.07E-07	4.48E-06	7.85E-02	6.37E-01	7.19E-01	9.53E-01	4.17E-02
**Carbohydrates**	1.26	1.40	1.62	0.80	0.75	0.73	0.85	0.83	2.82E-01	8.01E-02	8.11E-03	1.34E-01	3.45E-02	1.58E-02	2.56E-01	2.83E-01
**Amines**	4.26	2.01	1.30	1.38	0.98	1.19	1.17	1.33	2.59E-14	7.59E-11	1.35E-04	3.80E-05	6.30E-01	3.70E-03	3.11E-03	1.25E-04
**Indoles**	1.27	0.51	0.70	2.55	1.15	1.22	1.57	2.31	2.11E-01	1.76E-02	1.42E-01	7.59E-04	5.62E-01	1.56E-01	2.07E-02	6.28E-04
**Phenols**	1.18	1.89	1.70	0.96	1.10	0.96	1.39	1.08	4.82E-01	7.22E-02	1.08E-01	7.31E-01	4.36E-01	6.82E-01	2.21E-02	4.34E-01
**Steroids**	0.49	0.45	0.55	1.06	0.64	0.80	0.41	0.48	8.93E-02	6.79E-02	1.30E-01	7.84E-01	3.30E-02	2.12E-01	1.20E-03	9.41E-04
**Purines**	0.82	1.13	1.29	1.01	0.95	0.65	0.99	0.90	4.00E-01	5.67E-01	2.12E-01	9.53E-01	7.81E-01	2.74E-03	9.67E-01	5.06E-01
**Pyridines**	1.12	1.71	1.48	1.13	1.02	1.04	1.12	1.11	3.04E-01	1.79E-06	1.05E-04	1.57E-01	8.39E-01	6.77E-01	1.17E-01	2.15E-01
**Peptides**	1.88	1.71	1.57	1.10	1.56	1.18	1.16	1.05	5.41E-03	6.33E-02	6.21E-02	5.86E-01	3.64E-02	2.49E-01	4.02E-01	7.64E-01
**Others**	1.94	1.57	1.49	0.93	1.03	0.88	0.88	0.73	5.12E-03	6.19E-03	3.67E-03	3.60E-01	7.11E-01	8.44E-02	2.29E-01	3.73E-04

Fold change indicates the concentration of metabolites in corresponding week compared to W7. >1 means higher concentration compared to W7, and <1 means lower concentration compared to W7.

*P* value in Mann Whitney test indicates the statistical difference between the concentration of metabolites in corresponding week and W7. *P* < 0.05 indicates there is statistically significant difference between two groups.

**Table 2:** The metabolite variations between any two of the three brain regions at each time point (N = 6 each group).

**Metabolite Types**	**W7**			**W9**			**W12**			**W24**			**W56**			**W111**		
	**C/H**	**T/H**	**T/C**	**C/H**	**T/H**	**T/C**	**C/H**	**T/H**	**T/C**	**C/H**	**T/H**	**T/C**	**C/H**	**T/H**	**T/C**	**C/H**	**T/H**	**T/C**
**Lipids**	0.90	0.99	1.10	1.06	1.02	0.96	0.97	1.04	1.07	1.08	0.99	0.92	0.92	0.97	1.06	0.91	0.98	0.98
Glycerophospholipids	0.88	0.99	1.12	1.08	1.04	0.97	0.96	1.03	1.07	1.09	0.99	0.91	0.92	0.97	1.05	0.90	0.97	0.97
Sphingolipids	0.99	1.01	1.01	1.04	1.01	0.97	1.11	1.10	0.99	0.99	0.99	1.00	0.91	0.96	1.05	0.96	0.98	0.98
**Acylcarnitine**	1.11	1.06	0.95	1.11	1.14	1.03	1.16	1.18	1.02	1.14	0.93	0.82	0.78*^2^	0.83	1.06	0.91	1.20	1.20*^5^
**Free fatty acids**	1.14	1.07	0.94	1.00	0.97	0.97	0.97	0.95	0.97	1.22	1.00	0.82	0.86	0.99	1.15	1.08	0.90	0.90
Saturated fatty acids	1.12	1.05	0.93	0.99	0.95	0.95	0.95	0.93	0.97	1.16	0.99	0.85	0.84	0.97	1.15	1.04	0.89	0.89
Monounsaturated fatty acids	1.12	1.03	0.92	1.00	1.00	1.00	0.94	0.99	1.06	1.31	0.96	0.74	0.91	1.04	1.15	1.13	0.89	0.89
Polyunsaturated fatty acids	1.20	1.17	0.98	1.00	0.96	0.96	1.04	0.94	0.90	1.23	1.06	0.86	0.82	0.97	1.17	1.09	0.93	0.93
**Bile acids**	1.36	3.22	2.37	0.72	1.00	1.38	1.65	1.27	0.77	1.09	1.02	0.94	1.65	0.67	0.41	0.85	0.98	0.98
Unconjugated bile acids	0.82	3.24	3.97	0.73	1.04	1.42	0.90	1.09	1.22	1.11	1.10	0.99	0.65	0.76	1.16	0.82	0.97	0.97
Glycine conjugated bile acids	1.61	1.88	1.17	0.54	0.89	1.63	1.57	1.14	0.73	0.69	0.74	1.07	1.38	0.63	0.46	0.77	0.88	0.88
Taurine conjugated bile acids	4.44	5.44	1.22	1.15	1.03	0.89	4.65	2.13	0.46	1.99	1.29	0.65	4.86	0.46	0.09	1.16	1.26	1.26
**Amino acids**	1.04	1.00	0.96	0.98	1.08	1.10	1.10	1.04	0.95	1.06	0.95	0.90	0.91	1.01	1.10	0.95	1.04	1.04
**Organic acids**	1.01	0.95	0.94	0.96	0.93	0.97	1.02	1.14	1.12	1.15	1.12	0.97	0.95	0.91	0.96	0.91	0.85	0.85
**Carbohydrates**	0.59	0.70	1.20	1.23	1.15	0.94	0.93	1.04	1.11	1.05	0.98	0.93	0.72	0.81	1.14	0.93	0.86	0.86
**Amines**	0.99	0.95	0.96	0.93	1.18	1.27	1.02	1.03	1.01	0.90	0.98	1.09	0.84*^3^	1.05	1.26*^4^	0.96	0.99	0.99
**Indoles**	1.53	1.10	0.72	0.67	1.42	2.12	0.95	0.62	0.65	0.88	0.74	0.85	1.13	1.29	1.14	1.39	1.19	1.19
**Phenols**	0.90	1.00	1.11	0.81	0.88	1.09	0.92	0.92	1.00	1.09	1.08	0.99	0.98	0.94	0.95	0.89	0.95	0.95
**Steroids**	1.05	0.75	0.71	1.11	0.84	0.76	0.96	1.02	1.06	1.14	1.25	1.10	1.02	1.11	1.09	0.95	1.25	1.25
**Purines**	0.92	1.05	1.15	1.14	0.89	0.78	1.03	1.11	1.08	1.01	1.03	1.02	0.81	0.82	1.01	1.15	1.10	1.10
**Pyridines**	1.13	0.93	0.82	0.86	1.09	1.26	1.02	0.81	0.79	0.91	0.80	0.88	1.04	1.22	1.17	1.07	1.05	1.05
**Peptides**	0.63*^1^	0.95	1.50	0.92	1.25	1.36	0.73	0.76	1.05	0.77	0.67	0.88	1.24	0.91	0.74	1.02	1.36	1.36
**Others**	0.99	1.02	1.03	1.01	1.04	1.02	0.99	1.03	1.05	0.98	0.98	1.00	0.90	0.81	0.90	1.09	0.95	0.95

C indicates cortex; H indicates hippocampus; T indicates thalamus.

*^1^indicates *P* = 0.025, *^2^indicates *P* = 0.025, *^3^indicates *P* = 0.010, *^4^indicates *P* = 0.037, and *^5^indicates *P* = 0.036 in Mann Whitney test.
