# Supplementary material for: The Brain Metabolome of Male Rats across the Lifespan
Source: Sci Rep. 2016 Apr 11;6:24125. doi: 10.1038/srep24125 (PMC4827083; doi:10.1038/srep24125)
Supplement: Supplementary Information [file srep24125-s1.doc]

**SUPPLEMENTARY INFORMATION**

**The Brain Metabolome of Male Rats across the Lifespan**

**Xiaojiao Zheng^1^, Tianlu Chen^1^, Aihua Zhao^1^, Xiaoyan Wang^2^, Guoxiang Xie^3^, Fengjie Huang^1^, Jiajian Liu^1^, Qing Zhao^1^, Shouli Wang^1^, Chongchong Wang^2^, Mingmei Zhou^4^,** **Jun Panee^3^, Zhigang He^5^ & Wei Jia^1,3,4^,***

^1^Shanghai Key Laboratory of Diabetes Mellitus and Center for Translational Medicine, Shanghai Jiao Tong University Affiliated Sixth People’s Hospital, Shanghai 200233, China.

^2^Ministry of Education Key Laboratory of Systems Biomedicine, Shanghai Center for Systems Biomedicine, Shanghai Jiao Tong University, Shanghai 200240, China

^3^University of Hawaii Cancer Center, Honolulu 96813, USA.

^4^Center for Chinese Medical Therapy and Systems Biology, E-Institute, Shanghai University of Traditional Chinese Medicine, Shanghai 201203, China

^5^F. M. Kirby Neurobiology Center, Children’s Hospital, and Department of Neurology, Harvard Medical School, Boston, MA 02115, USA.

*Correspondence and requests for materials should be addressed to W.J. (email: wjia@cc.hawaii.edu).

**Supplementary Methods**

**Principle Variance Component Analysis (PVCA)**

PVCA estimates the magnitude of each source of variability and visually compares standardized variance components estimates. This strategy involves four basic steps: (1) perform PCA to reduce the dimension and retain the majority of the variability in the expression data, say with the first few principal components; (2) fit a mixed model separately to each principal component with all factors of interest as random effects and any nuisance factors as fixed effects; (3) for each factor, average the estimated variance components with their corresponding eigenvalues as weights; (4) standardize the weighted average variance components estimates by dividing by their sum, so that the magnitude of each effect can be represented as a proportion of the total variance.

PVCA has several appealing features. First, the modeling procedure is effectively invariant to the order of the factors. Thus, any potential confounding issue between factors will typically not interfere with the variance estimation, as all factors compete to explain variability. Second, the variance component for each factor will be estimated through restricted maximum likelihood (REML), which is the most efficient and accurate means to estimate variance components, especially when an unbalanced experimental design is involved. Third, variance components analysis (VCA) can also be utilized to estimate the model residual, providing a convenient way to summarize unexplained variance and assess its magnitude against all other known factors in the model.

**Metabolic Data Calibration Tool (MDCT)**

MDCT is a series of in house matlab code with user-friendly GUI for metabolic data normalization and pretreatment. Here, we used this tool to pretreat pooled sample quality controls (PQCs) and normalized sample data using the ‘window’ mode. This mode is recommended for smoothing out short-term fluctuations and highlighting long-term trends. Window size was set to 3, RSD ratio was set to 15%, and missing value ratio was set to 0.1. Supplementary Fig. 7 is the flowcharts of the MDCT and PQC pretreatment workflow.

**Principal Component Analysis (PCA)**

PCA is an conventional unsupervised multivariate analysis method aiming for dimension reduction. This technique transforms an original set of correlated variables to a new set of uncorrelated variables, called principal components (PCs). This transformation is defined in such a way that the first principal component has the largest possible variance (that is, accounts for as much of the variability in the data as possible), and each succeeding component in turn has the highest variance possible under the constraint that it is orthogonal to the preceding components. The scores of PCs are coordinates of observations (samples in this report) when it is projected on the projection plane or hyper plane of a model.

**Partial Least Squares Projection to Latent Structures regression (PLS)**

PLS is a kind of supervised multivariate analysis method bearing some relation to principal components regression. It finds the linear (or polynomial) relationship between a matrix Y (age and region in this report) and a matrix X (all the identified metabolites). This modeling geometrically corresponds to fitting a line, plane or hyper plane to both the X and Y data represented as points in a multidimensional space, with the objective of well approximating the original data tables X and Y, and maximizing the covariance between the observation positions on the hyper planes. The scores plot of PLS model displays the situation of observations (samples in this report) on the projection plane or hyper plane of a model.

**PLS Discriminate Analysis (PLS-DA)**

PLS-DA, a special case of PLS, is a PLS regression for classes discrimination with a dummy Y variable (i.e. a grouping variable).

**The correlation analysis of bile acids and carbohydrate metabolism or tricarboxylic acid cycle**

The total concentrations of bile acids and three subtypes were correlated with the first principal component scores of PCA derived from the metabolites in carbohydrate metabolism or tricarboxylic acid cycle by calculating their Spearman’s correlation coefficients in SPSS. A total of 22 metabolites were detected in carbohydrate metabolism, including glucose, glucose-6-phosphate, pyruvic acid, ribose, glycerol, ascorbic acid, dehydroascorbic acid, glyceraldehydes, threitol, 1,5-anhydro-d-sorbitol, adonitol, hexose, glucitol, N-acetylglucosamine, galactose, fructose, ribonic acid, glycerol phosphate, threonic acid, myo-inositol, lactose, and arabitol. Five metabolites were detected in tricarboxylic acid cycle, including pyruvic acid, succinic acid, fumaric acid, citric acid and malic acid.

**
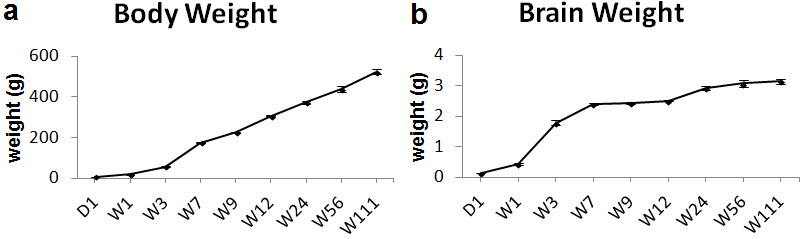
**

**Supplementary Figure S1. The body weight (a) and brain weight (b) of the rats at the time of sacrifice.** The error bars indicate standard errors. N = 6 per group.


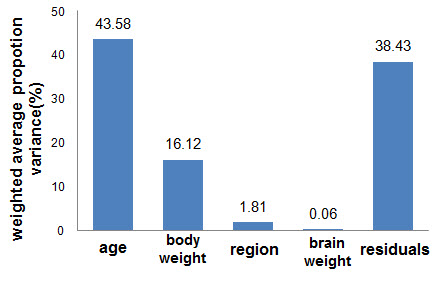


**Supplementary Figure S2. Weighted average proportion variance explained by age, body weight, region, brain weight, and individual effects in metabolite data of W7 to W111.** The normalized metabolite data with 380 detected metabolites from the six time points were used in the principal component analysis. Principal components with substantial contribution to the total variance greater than 10% were used as response variables to fit a mixed linear model with different source of variability (age, body weight, region, and brain weight) as random effects. The model was fitted via restricted maximum likelihood (REML) and was used to obtain the variance component estimates. The weighted average variance was then calculated based on the eigenvalues retained from the principal component analysis. In total, the first 3 principal components with combined contribution to the total variance of 67.2% were used.


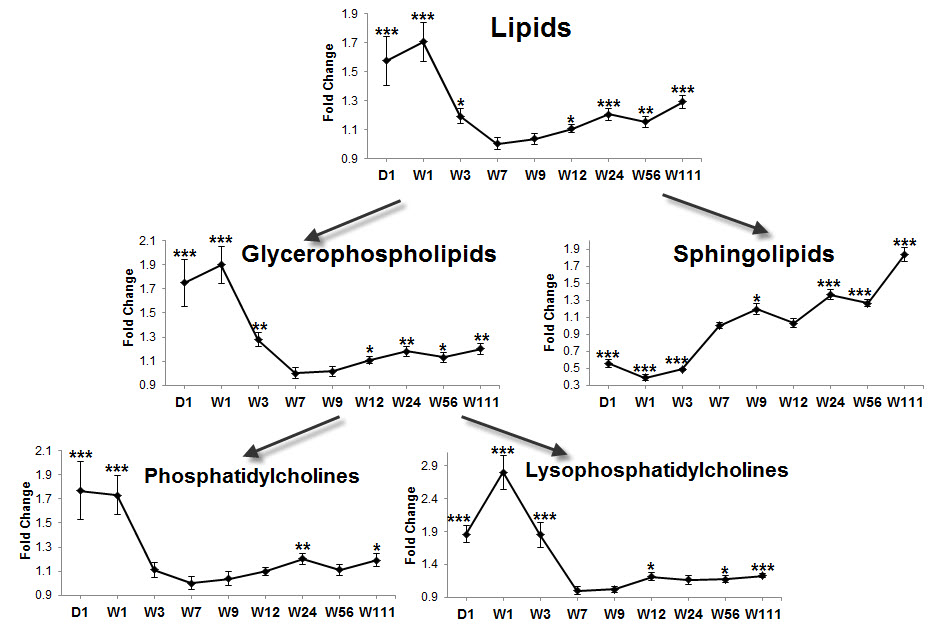


**Supplementary Figure S3. Dynamic alteration of lipids and their subtypes across lifespan.** The line charts show the fold changes (y axis) of lipids and their subtypes at each time point in comparison to that at W7 (x axis). The error bars indicate standard errors. N = 6 each group. * indicates *P* < 0.05, ** indicates *P* < 0.01, and *** indicates *P* < 0.001 in Mann Whitney test compared to W7.


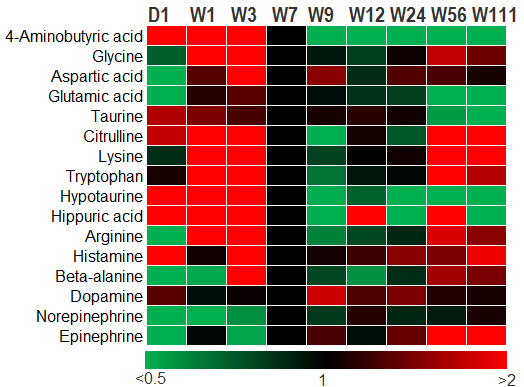


**Supplementary Figure S4. Heatmap of neurotransmitters.** Each cell in the heatmap represents the fold change of the concentration at the time point noted above the map in comparison to that of W7. The color key is shown below the map.


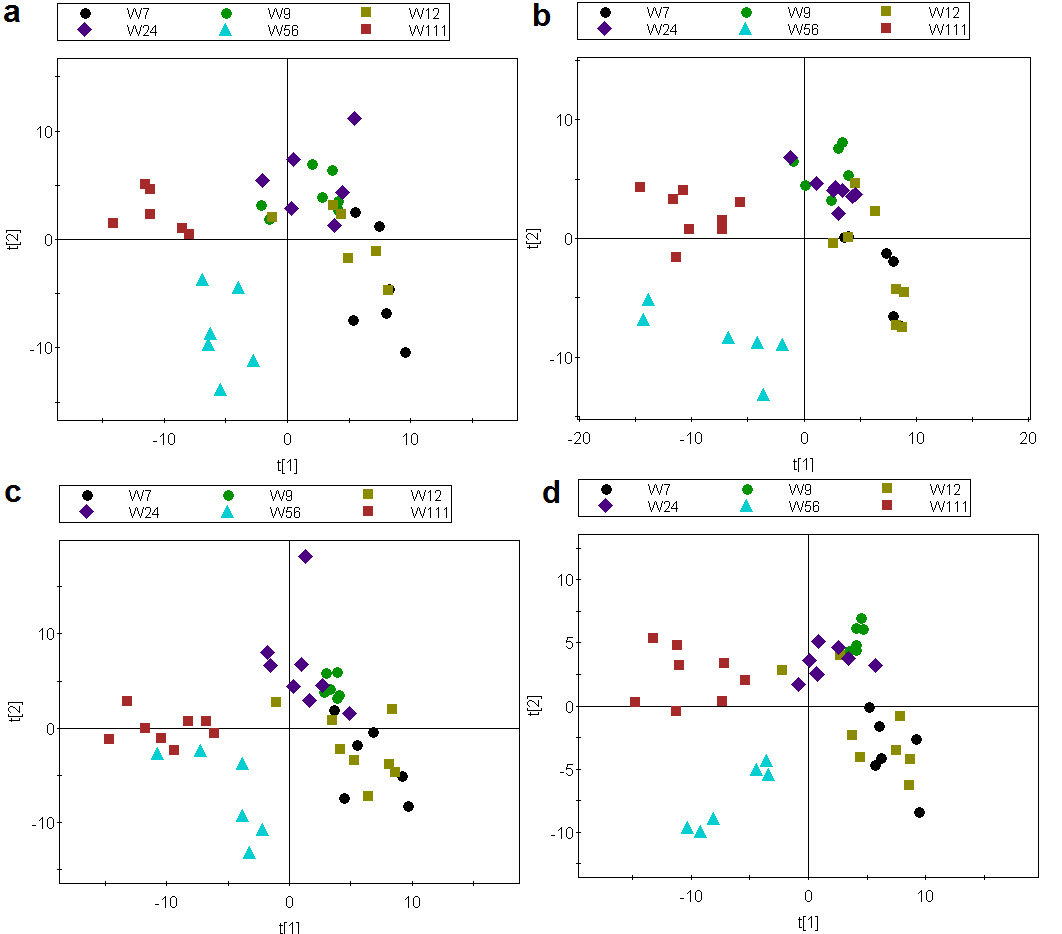


**Supplementary Figure S5. PLS-DA scores plot of metabolomes of the whole brain and three anatomical regions.** (a) PLS-DA scores plot of the whole brain; (b) PLS-DA scores plot of the hippocampus; (c) PLS-DA scores plot of the cortex; (d) PLS-DA scores plot of the thalamus.


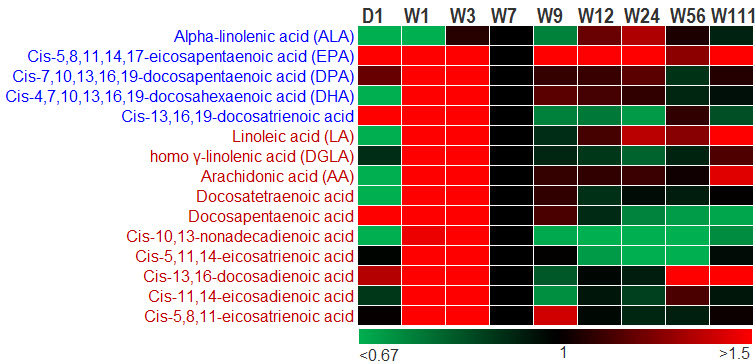


**Supplementary Figure S6. Heatmap of omega-3 (blue words) and omega-6 (red words) fatty acids.** Each cell in the heatmap represents the fold change of the concentration at the time point noted above the heatmap in comparison to that of W7. Color key is shown below the heatmap.


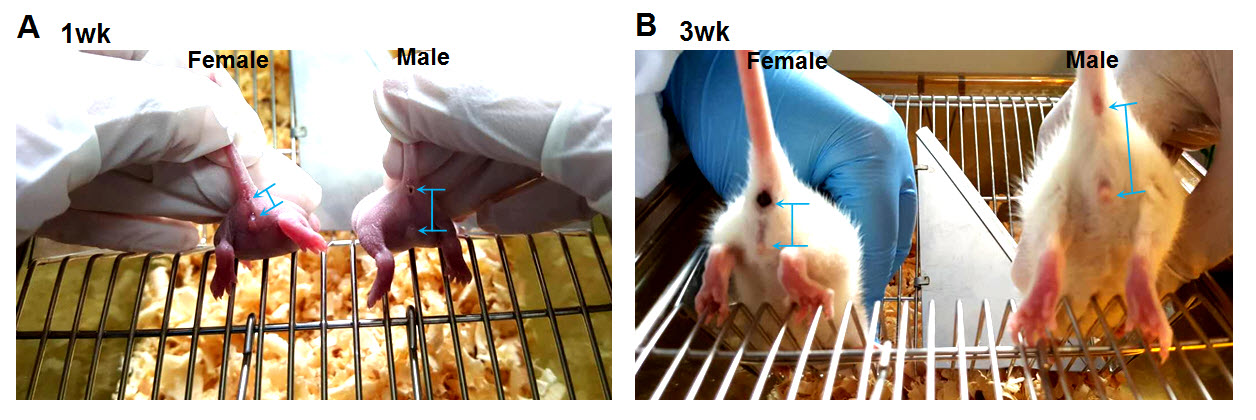


**Supplementary Figure S7.** (A) One-week old Wistar rats; (B) Three-week old Wistar rats. The blue arrows point the anus and the genital papilla, respectively.


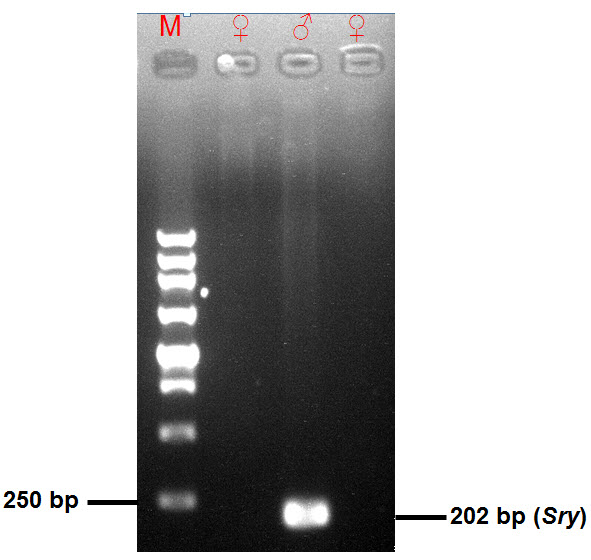


**Supplementary Figure S8. Example of PCR-based sex identification in three rats, using PCR amplification of male-specific *Sry* gene.**


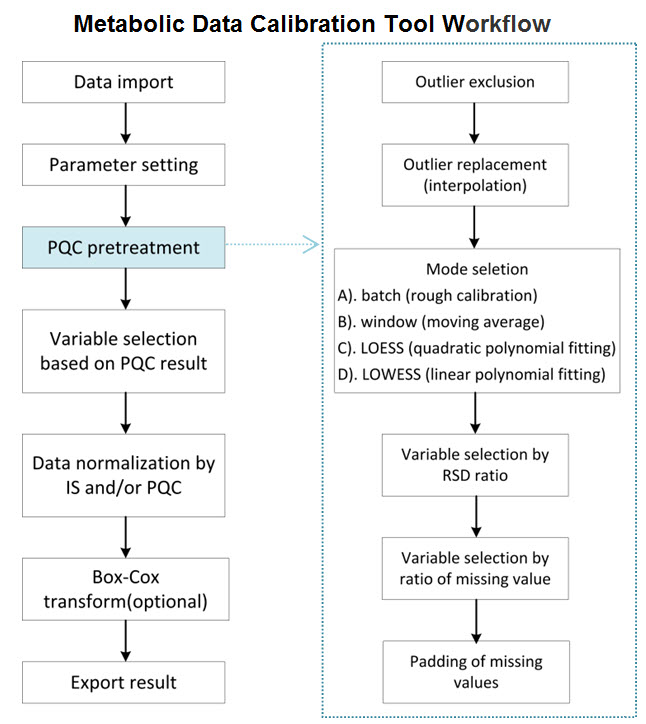


**Supplementary Figure S9. Flowcharts of the metabolic data calibration tool workflow.**

**Supplementary Table S1. Summary of detected metabolite types and subtypes.**

| **Metabolite types** | **Number of identification** | **Number of quantitation** | **Subtypes** |
| --- | --- | --- | --- |
| Lipids | 101 | 99 | Glycerophospholipids (87) |
|  |  |  | Sphingolipids (14) |
| Acylcarnitines | 41 | 40 |  |
| Free fatty acids | 40 | 40 | Saturated fatty acids (15) |
|  |  |  | Monounsaturated fatty acids (10) |
|  |  |  | Polyunsaturated fatty acids (15) |
| Bile acids | 20 | 20 | Unconjugated bile acids (9) |
|  |  |  | Glycine conjugated bile acids (4) |
|  |  |  | Taurine conjugated bile acids (7) |
| Amino acids | 63 | 25 |  |
| Organic acids | 20 |  |  |
| Carbohydrates | 26 | 1 |  |
| Amines | 11 | 5 |  |
| Indoles | 5 | 1 |  |
| Phenols | 6 | 1 |  |
| Steroids | 3 |  |  |
| Purines | 12 |  |  |
| Pyridines | 8 |  |  |
| Peptides | 6 |  |  |
| Others | 18 |  |  |

**Supplementary Table S2. The correlation results of bile acids and carbohydrate metabolism or tricarboxylic acid cycle**

|  | **Total bile acids** | **Unconjugated bile acids** | **Glycine conjugated bile acids** | **Taurine conjugated bile acids** |
| --- | --- | --- | --- | --- |
| **Carbohydrate Metabolism** | R = 0.116  P = 0.402 | R = 0.328  P = 0.016 | R = -0.106  P = 0.444 | R = -0.296  P = 0.033 |
|  |  |  |  |  |
| **Tricarboxylic acid cycle** | R = -0.175  P = 0.204 | R = - 0.085  P = 0.543 | R = -0.476  P = 0.00027 | R = -0.064  P = 0.647 |

R indicates Spearman’s correlation coefficient.

P indicates P-value with *P* < 0.05 set as the level of statistical significance.
